# Atorvastatin does not protect against ischemia-reperfusion damage in cholestatic rat livers

**DOI:** 10.1186/s12893-017-0235-9

**Published:** 2017-04-11

**Authors:** Jimme K. Wiggers, Rowan F. van Golen, Joanne Verheij, Annemiek M. Dekker, Thomas M. van Gulik, Michal Heger

**Affiliations:** 1 Department of Surgery, Surgical Laboratory, Academic Medical Center, University of Amsterdam, Amsterdam, The Netherlands; 2 Department of Pathology, Academic Medical Center, University of Amsterdam, Amsterdam, The Netherlands

## Abstract

**Background:**

Extrahepatic cholestasis sensitizes the liver to ischemia/reperfusion (I/R) injury during surgery for perihilar cholangiocarcinoma. It is associated with pre-existent sterile inflammation, microvascular perfusion defects, and impaired energy status. Statins have been shown to protect against I/R injury in normal and steatotic mouse livers. Therefore, the hepatoprotective properties of atorvastatin were evaluated in a rat model of cholestatic I/R injury.

**Methods:**

Male Wistar rats were subjected to 70% hepatic ischemia (during 30 min) at 7 days after bile duct ligation. Rats were randomized to atorvastatin treatment or vehicle-control in three test arms: (1) oral treatment with 5 mg/kg during 7 days after bile duct ligation; (2) intravenous treatment with 2.5, 5, or 7.5 mg/kg at 24 h before ischemia; and (3) intravenous treatment with 5 mg/kg at 30 min before ischemia. Hepatocellular damage was assessed by plasma alanine aminotransferase (ALT) and histological necrosis.

**Results:**

I/R induced severe hepatocellular injury in the cholestatic rat livers (~10-fold increase in ALT at 6 h after I/R and ~30% necrotic areas at 24 h after I/R). Both oral and intravenous atorvastatin treatment decreased ALT levels before ischemia. Intravenous atorvastatin treatment at 5 mg/kg at 24 h before ischemia was the only regimen that reduced ALT levels at 6 h after reperfusion, but not at 24 h after reperfusion. None of the tested regimens were able to reduce histological necrosis at 24 h after reperfusion.

**Conclusion:**

Pre-treatment with atorvastatin did not protect cholestatic livers from hepatocellular damage after I/R. Clinical studies investigating the role of statins in the protection against hepatic I/R injury should not include cholestatic patients with perihilar cholangiocarcinoma. These patients require (pharmacological) interventions that specifically target the cholestasis-associated hepatopathology.

## Background

Liver surgery in patients with perihilar cholangiocarcinoma is associated with a high rate of postoperative liver failure and related mortality (between 5 to 18%) because these tumors obstruct bile flow preoperatively [1–4]. The resultant cholestasis afflicts patients’ systemic and liver condition. One effect that especially sensitizes patients to postoperative liver failure is the increased susceptibility of cholestatic livers to ischemia/reperfusion (I/R) injury.

Hepatic I/R injury results from the temporary deprivation of blood supply to the liver, which is used to prevent excessive peri-operative blood loss. Reoxygenation of the liver after resection causes overproduction of reactive oxygen and nitrogen species (ROS and RNS, respectively) [5]. and subsequently induces mainly necrotic cell death [6]. Dying and dead hepatocytes release numerous endogenous molecules that act as damage-associated molecular patterns (DAMPs), which attract neutrophils that afflict liver microvasculature and activate Kupffer cells to produce more ROS. These processes altogether increase intrahepatic oxidative stress, and cause (micro)vascular constriction and inflammation [7]. Cholestasis exacerbates hepatic I/R injury due to the pre-existent hepatopathology [8]. The accumulation of hydrophobic bile acids in cell and organelle (mitochondrial) membranes leads to increased mitochondrial ROS/RNS production, sterile inflammation, and cell death [9–11]. In addition, it is characterized by microvascular perfusion defects [12]. Together, cholestasis leads to an impaired energy status and overall metabolic dysfunction that is vulnerable to I/R [8].

**Fig. 1:**
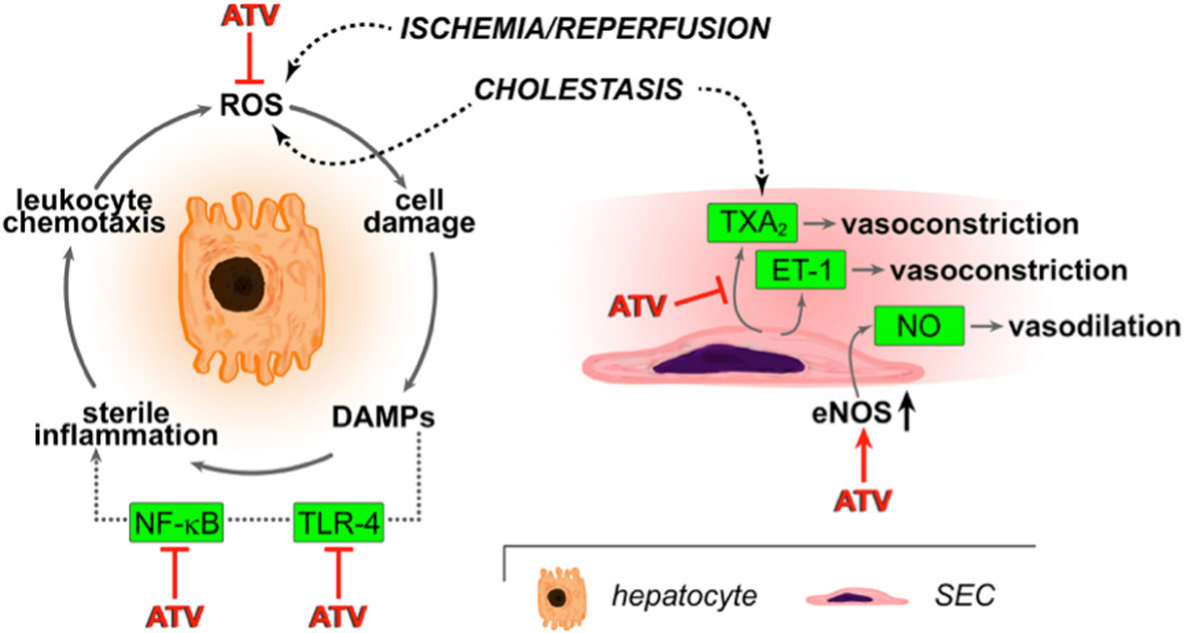
Pathophysiological mechanisms in hepatocytes and sinusoidal endothelial cells (SEC) as a result of ischemia/reperfusion and cholestasis in the context of the pharmacodynamics of atorvastatin (ATV). The sites where ATV has been demonstrated to intervene are indicated and serve as a basis for this study. Abbreviations: ROS, reactive oxygen species; DAMPs, damage-associated molecular patterns; TLR-4, Toll-like receptor-4; NF-κB, nuclear factor kappa-light-chain-enhancer of activated B cells; TXA_2_, thromboxane A2; ET-1, endothelin-1; NO, nitric oxide; eNOS, endothelial nitric oxide synthase; SEC, sinusoidal endothelial cell

Stains are normally used in the prevention of cardiovascular disease because of their lipid-lowering properties. but also have pleiotropic effects [13]. These drugs have been shown to reduce markers of liver injury in animal models of cholestasis [14–16]. Although these effects remain to be confirmed in clinical studies, statins seem to reduce bile acid production and increase cholesterol transport back to plasma; both regulated by nuclear receptors [17, 18]. Moreover, statins have previously been reported to protect against I/R injury in normal and steatotic mouse livers. The supposed underlying mechanisms of statin protection include antioxidant, [19], vasoprotective, [20–25] anti-inflammatory, [20, 26] and anti-thrombotic effects [20, 21, 27, 28]. In that respect, the dual hepatoprotective properties of statins, as outlined in Fig. 1, may prove especially helpful against I/R injury in the context of cholestatic hepatopathology.

The aim of this study was to determine whether preoperative atorvastatin (ATV) treatment could pharmacologically reduce injury before and after I/R in cholestatic livers. This was tested in a bile duct ligation rat model of obstructive cholestasis, which is representative of patients requiring liver surgery for perihilar cholangiocarcinoma.

## Methods

### Animals

Specific pathogen-free male Wistar rats (*N* = 115, Harlan Laboratories, Horst, the Netherlands) weighing between 250–270 g were acclimated for 1 week in a temperature-controlled room with 12-h dark/light cycles and ad libitum access to water and standard chow.

### Anesthesia

For surgical procedures, rats were anesthetized with 3–5% isoflurane (O_2_:air ratio of 1:1, 2 L/min, Forene, Abbott Laboratories, Queensborough, UK) and analgesic care was provided by subcutaneous administration of buprenorphine (0.03 mg/kg, Temgesic, Schering-Plough, Kenilworth, NJ). Maintenance anesthesia comprised 2–2.5% isoflurane (O_2_:air ratio of 1:1, 1 L/min).

### Surgical procedures

Standard bile duct ligation (BDL) was used to induce cholestasis. The liver was exteriorized after a midline laparotomy, and the common bile duct was ligated twice and dissected between the ligatures. Seven days after BDL, rats underwent a re-laparotomy and the liver was again exteriorized. A non-traumatic vascular sling was then placed around the afferent vessels (arterial and portal vessels) to the median and left lateral lobes to induce ±70% hepatic ischemia for 30 min [8].

### Atorvastatin preparation

For oral administration, ATV (Pfizer, New York, NY) was dissolved in sterile 0.9% NaCl solution (B. Braun Melsungen, Melsungen, Germany) at a 1.0 mg/mL concentration. For intravenous (i.v.) administration, atorvastatin (PZ0001, Sigma-Aldrich, St. Louis, MO) was dissolved in dimethyl sulfoxide (DMSO) at a 10.0 mg/mL stock concentration. Systemically dosed ATV was administered via the tail vein.

### Experimental design

**Fig. 2:**
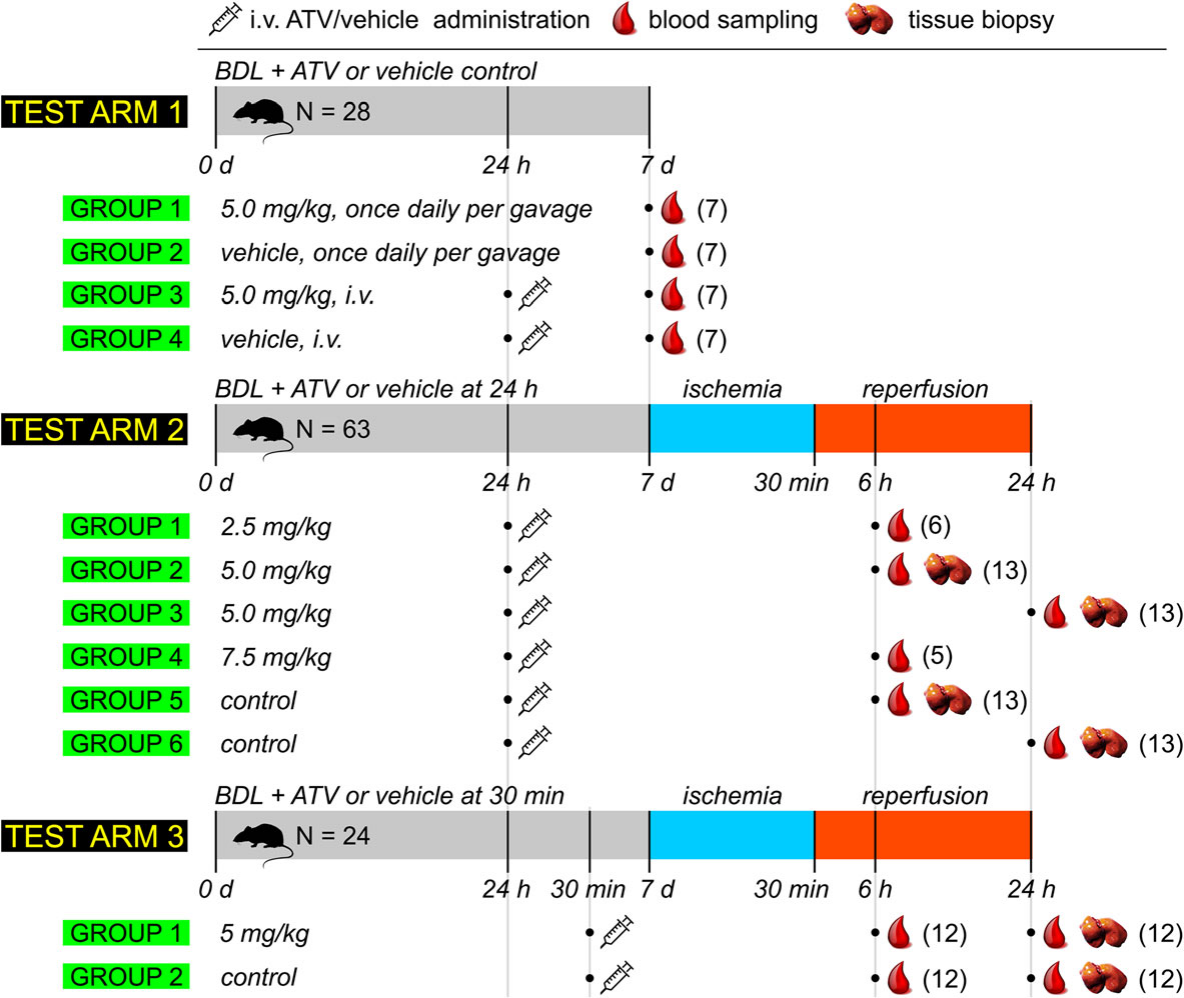
Experimental setup consisting of 3 test arms. The first test arm entailed oral or intravenous (i.v.) administration of atorvastatin (ATV) without subjecting the animals to ischemia/reperfusion (I/R) to determine the effect of ATV on the pathology of cholestasis. This test arm also served to demonstrate that ATV is targeted to the liver following oral or i.v. administration. Test arms 2 and 3 encompassed i.v. administration of ATV followed by I/R. The gray segment delineates the period of bile duct ligation (BDL), the blue segment indicates the ischemic phase, and the red segment designates the reperfusion time frame. The times in in every segments at which procedures were performed are indicated with black vertical lines, protracted in gray throughout the figure. The legend at the top explains the significance of the symbols, the numbers in parentheses refer to the group size

Rats were randomized to ATV treatment or vehicle-control in three test arms according to Fig. 2. Randomization consisted of drawing folded sheets of paper with written treatment assignment out of a closed bag for each animal.

#### Test arm 1

In the first test arm, the effects of orally and i.v. administered ATV were investigated in the context of cholestatic liver injury before ischemia. ATV (or its vehicle control) was administered per gavage once daily during 7 d after BDL at a dose of 5.0 mg/kg body weight. For i.v. administration, the ATV 10.0-mg/mL stock concentration was diluted with NaCl to a concentration of 1.0 mg/mL, corresponding to an ATV dose of 5.0 mg/kg body weight. Blood samples were collected before ischemia but animals were not sacrificed at this stage, because some of the animals in test arm 1 subsequently underwent I/R and continued the experiment as part of test arm 2.

#### Test arm 2

In the second test arm, the effect of a single i.v. dose of ATV administered 24 h before ischemia induction was investigated. First, a dose finding study was used to determine the most optimal dosing regimen based on ALT levels at 6 h after reperfusion (groups 1, 2, and 4 according to Fig. 2). For this purpose, ATV in DMSO (10.0 mg/mL) was diluted with NaCl to a concentration of 0.5, 1.0, or 1.5 mg/mL, corresponding to administered doses ATV of 2.5, 5.0, or 7.5 mg/kg body weight. Secondly, the most optimal dosing regimen was tested in expanded groups, and animals in these expanded groups were sacrificed at 6 and 24 h after reperfusion.

#### Test arm 3

In the third test arm, the effect of a single i.v. dose of ATV administered 30 min before ischemia induction was investigated in terms of I/R-induced liver injury at 6 and 24 h reperfusion.

#### Group sizes

All groups were started with a minimum size of *n* = 5–7 animals. In test arm 1, group sizes were limited to *n* = 7 after results had sufficiently reached the threshold for significance. In test arm 2, preliminary analysis with 5–7 animals in all groups was used to choose the most optimal dosing regimen. Subsequently, groups with the most optimal dosing regimen were expanded according to power analysis, which was based on the preliminary analysis. An expected difference of 30% in histologic necrosis at 6 and 24 h reperfusion resulted in minimum group sizes of *n* = 12 (standard deviation 25%, α = 0.05, β = 0.8). Group sizes in test arm 3 were based on the same power analysis.

### Sample collection

Blood sampling was performed via the tail vein when the animals remained in the experiment (test arm 3) or via cardiac puncture when the animals were sacrificed by exsanguination. Following sacrifice, liver specimens were harvested for histological processing. Standard biopsies were taken of the liver lobes that had been subjected to ischemia. This included one biopsy of the center of the median lobe, and one biopsy of the center of the left lateral lobe. For each animal, the average result of these two biopsies determined the final result that was included in statistical analysis.

### Assessment of liver injury, inflammation, and fibrosis

Serum ALT and bilirubin levels were assayed in blood samples (Fig. 2) by routine clinical chemistry using a Cobas 8000 modular analyzer (Roche, Basel, Switzerland). Histological sections were processed as described previously and stained with hematoxylin and eosin (H&E) [29].

The extent of necrosis was quantified by an experienced hepatopathologist (JV) in liver biopsies collected after I/R (Fig. 2). Analysis was performed in 10 random fields of view (FOVs) per liver and expressed as a percentage of the total FOV surface [30].

### Statistical analysis

Statistical analysis was performed in GraphPad Prism (GraphPad Software, San Diego, CA). Results are presented as mean ± SEM. ATV and vehicle control-treated groups were compared using an unpaired student’s *t*-test with Welch’s correction. The Gaussian distribution of each data set was confirmed with a Shapiro-Wilk test (*n* < 8) or a D’Agostino-Pearson omnibus test (*n* ≥ 8). A *P*-value of ≤ 0.05 was considered statistically significant.

## Results

**Fig. 3:**
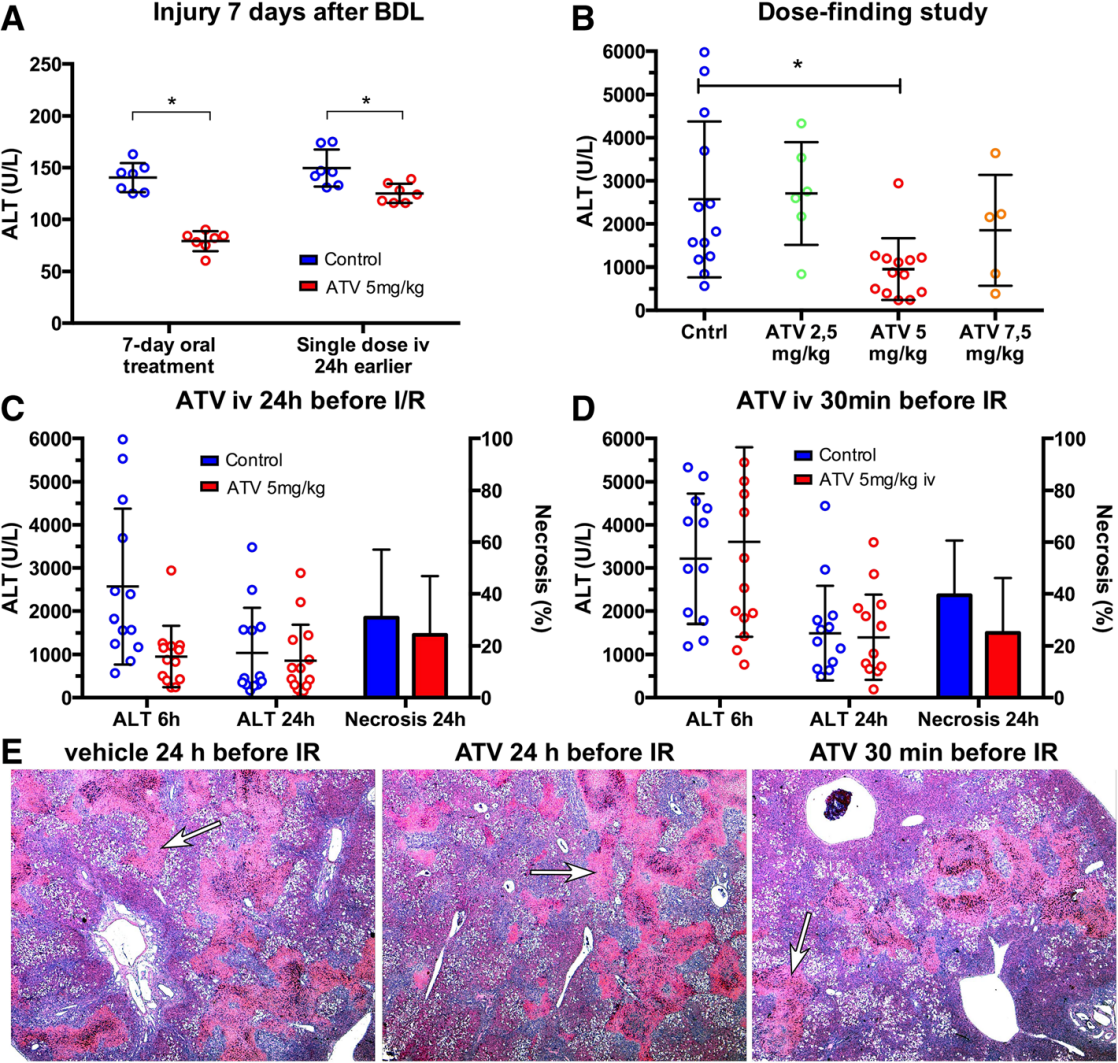
Study results. (**a**) Oral and single-dose i.v. Atorvastatin treatment reduced serum ALT at 7 d after bile duct ligation. (**b**) Atorvastatin 5 mg/kg administered 24 h before I/R was identified as the most effective dose at reducing serum ALT 6 h after I/R. (**c**) Despite its protective capacity at 6 h after I/R, atorvastatin 5 mg/kg administered 24 h before I/R did not reduce serum ALT or necrosis at 24 h after I/R. (**d**) No effect in serum ALT or necrosis was observed when atorvastatin 5 mg/kg was administered 30 min before I/R. (**e**) Representative histological sections (H&E) of I/R-subjected cholestatic rat livers treated by vehicle or ATV at 24 h or 30 min before the induction of ischemia. The white arrows point to patches of confluent necrosis. Original magnification 2.5 ×

The 7-days BDL resulted in severe cholestasis, as evidenced by a mean total bilirubin of 174 ± 23 μM. Histological sections acquired after I/R exhibited septal fibrosis, which had developed before I/R due to the chronic nature of this process. Cholestasis was also associated with elevated plasma ALT levels (Fig. 3a), reflecting hepatocellular injury that was consistent with previous reports [8].

Daily oral doses of 5 mg/kg ATV during the 7-days BDL period as well as a single i.v. dose of 5 mg/kg on day 6 during the 7-days BDL period (test arm 1) reduced ALT levels by ~50% (140 ± 5 U/L for control vs. 79 ± 4 U/L for ATV) and ~20% (150 ± 7 U/L for control vs. 125 ± 4 U/L for ATV), respectively (Fig. 3a). These data indicate that ATV (a) reached the liver following oral as well as systemic administration and (b) conferred a hepatoprotective effect during the progression of cholestatic liver injury.

Inasmuch as cholestatic hepatopathology is amplified as a result of I/R, the pharmacodynamic efficacy of ATV was examined in a dose finding setting at 6 h reperfusion following 30 min of ischemia. As shown in Fig. 3b, i.v. administration of ATV at 5 mg/kg 24 h before the induction of ischemia resulted in significantly reduced ALT levels at 6 h reperfusion (2572 ± 500 U/L for control vs. 953 ± 197 U/L for ATV; *P* = .01). Consequently, this dosing regimen was more closely investigated in terms of hepatocellular damage (ALT) and histological necrosis. The other dosing regimens at 24 h before ischemia induction were not able to significantly decrease ALT levels at 6 h reperfusion (Fig. 3b).

Although ATV 5 mg/kg i.v. 24 h before ischemia reduced ALT levels at 6 h reperfusion, the protective effects were abrogated at 24 h reperfusion, as measured in terms of ALT levels (1036 ± 288 U/L for control vs. 859 ± 223 U/L for ATV; *P* = .63; Fig. 3c) and histological necrosis (31 ± 7% for control vs. 23 ± 6% for ATV; *P* = .43; Fig. 3c and e). Given that necrosis is irreversible, these results demonstrated that ATV was unable to ultimately protect the liver from damage when administered systemically 24 h before I/R.

It is known that ATV is taken up by sinusoidal endothelial cells and hepatocytes, [31] and metabolized in and excreted by the liver [32]. Administering ATV 24 h before I/R may therefore have resulted in subtherapeutic intrahepatic ATV levels, accounting for the absence of hepatoprotection. To resolve this potential pharmacokinetic hurdle, ATV was administered 30 min before ischemia induction at the optimal concentration (5 mg/kg) in accordance with previous reports (24). Nevertheless, this treatment regimen neither resulted in reduced liver damage at 24 h of reperfusion, as measured in terms of ALT levels (1491 ± 1094 U/L for control vs. 1396 ± 986 U/L for ATV; *P* = .91; Fig. 3d) and necrosis (39 ± 6% for control vs. 25 ± 6% for ATV; *P* = 0.12; Fig. 3d and e). In light of these data, subsequent experiments were discontinued, as there was no compelling evidence that ATV protects cholestatic liver from I/R injury.

## Discussion

Atorvastatin has been shown to protect against I/R injury in experimental models of normal and steatotic livers [20, 21, 27, 28]. Even short-term therapy with ATV (5 mg/kg) just 1 h before ischemia in normal and steatotic mouse livers conferred a 70–90% reduction in post-I/R necrosis [23]. Other studies used varying dosages of statin pretreatment at varying time points and found similar effects in reduction of hepatopathology; these regimens included ATV pretreatment 10 mg/kg 24 h and again 1 h before ischemia induction, [33] Simvastatin (5 mg/kg) pretreatment 1 h before ischemia induction, [28] and Simvastatin (1 mg/kg) even 30 min before ischemia induction [21]. Key mechanisms include suppression of inflammation and microvascular protection. Statins reduce activity of signaling proteins Toll-like receptor-4 and High mobility group box 1 (HMGB1), translating to reduced activity of downstream inflammation mediator nuclear factor kappa B (NF-κB) as well as cytokines tumor necrosis factor alpha (TNF-a) and interleukin 6 (IL-6) [20, 23, 33]. Statins also upregulate endothelial nitric oxide synthase (eNOS) production [21, 24]. eNOS stimulates the production and bioavailability of nitric oxide (NO) in the vascular endothelium, which subsequently causes vasodilatation of the hepatic microvasculature, and suppresses thromboxane A2 production [25, 28]. Another study found that vasoprotective effects of ATV were modulated through a decrease in intracellular adhesion molecule-1 (ICAM-1) and preservation of antithrombin-III (ATIII) levels [33]. These hepatoprotective mechanisms attributed to statins are schematically outlined in Fig. 2 for ATV. Next to anti-inflammatory and vasoprotective mechanisms, statins also exert anti-oxidant effects after I/R. One study found an increase in antioxidant enzyme activities after statin pretreatment, including superoxide dismutase (SOD), glutathione peroxidase (GPx) and catalase (CAT), [28] and another study found that antioxidant effects are also achieved by a reduction in nicotinamide adenine dinucleotide phosphate (NADPH) oxidase activity [19]. The above-presented results of previous studies warrant clinical trials that should test if statins can effectively be used to protect normal and steatotic livers against I/R injury in clinical practice.

We hypothesized that ATV would also protect cholestatic livers against I/R injury. ATV potentially intervenes in several of the processes that are associated with pre-existing liver damage in cholestasis, including sterile inflammation, [9–11] microvascular perfusion defects, [12, 34, 35] and impaired energy status [36]. The experiments demonstrated that oral ATV administered daily (5 mg/kg) as well as a single i.v. bolus 24 h before animal sacrifice (5 mg/kg) reduced the extent of cholestasis-induced liver damage during 7-d BDL, indicating that ATV reached the liver and conferred a hepatoprotective effect. However, systemic administration of ATV did not protect the liver against I/R damage, regardless of the dosage (up to 7.5 mg/kg) and time of administration (24 h or 30 min before ischemia induction). Further mechanistic elucidation was therefore not performed and the study was terminated prematurely.

Patients with perihilar cholangiocarcinoma typically present with obstructive jaundice and cholestasis upon admission, which constitute severe risk factors in liver surgery. Consequently, biliary drainage is often employed to alleviate these conditions prior to surgery. Nonetheless, some patients are selected to undergo surgery without biliary drainage, [3, 4] and these patients may benefit from statin treatment to reduce liver injury before surgery, based on our results and those of others [14–16, 37]. Similarly, patients with primary biliary cirrhosis may benefit from statin treatment. One report described lower total bile acid levels, [38] and several reports described a reduction in cholestasis markers in patients with primary biliary cirrhosis [38, 39]. It should be noted that recent studies did not reproduce these findings, [40, 41] so the actual clinical benefit of statins in the treatment of cholestasis remains unclear.

The exact reasons for the failure of ATV to prevent hepatocellular damage after I/R in cholestatic livers are currently elusive, and may be explained by several mechanisms. Microvascular perfusion defects and impaired energy status may have persisted after ATV treatment. This could have been caused by mechanical compression of the hepatic microcirculation due to biliary hyperdilatation as a result of the cholestasis, impairing intrahepatic blood flow and energy metabolism [8]. Alternatively or additionally, cholestasis may have caused a pre-existent vasoconstrictive state that rendered the hepatic microcirculation unreceptive to ATV-mediated inhibition of vasoconstrictors (endothelin-1, thromboxane A2) and upregulation of NO, which would generally result in improved microcirculation, oxygen delivery, and energy metabolism. Also, the poor responsiveness to ATV treatment may have been exacerbated by the prevailing state of oxidative stress and sterile inflammation in cholestatic livers, [8] which cannot be fully resolved by HMGB-1, TLR4 and NF-κB inhibition with ATV [20, 23]. In light of the negative results, we chose not to further investigate the mechanisms that underlie the lacking therapeutic efficacy of ATV. Instead, future research efforts should be directed at evaluating other types of pharmaceutical agents that target the multifarious pathogenic features of I/R injury in cholestatic livers.

Lastly, readers should note that this study has several limitations. First, the experimental model was associated with substantial variability in outcomes, despite the broad experience with this model in our laboratory [8, 42–44]. The group sizes had to be extended during the study following interim analysis to overcome the considerable standard deviations, but were still inadequate to statistically resolve minor beneficial effects of ATV. However, it is questionable whether such small improvements in outcome would justify the use of ATV as an intervention. Moreover, liver damage following I/R was extensive, probably owing to 7 days of BDL prior to ischemia induction. Although this is a widely used model for extrahepatic cholestasis, we cannot preclude that ATV does not protect the liver against I/R injury in cases of milder cholestasis. Lastly, BDL was irreversible in the model used in this study. Reconstruction of bile flow in the reperfusion phase would have better resembled the clinical situation of patients with perihilar cholangiocarcinoma, who normally undergo a bile duct reconstruction after partial liver resection (i.e. hepaticojejunostomy). Nonetheless, such an additional procedure would have prolonged laparotomy time, and as a consequence would have added even more variability to the model.

## Conclusions

Pre-treatment with Atorvastatin did not protect cholestatic rat livers from hepatocellular damage after I/R. Clinical trials are currently warranted to investigate if statins can ameliorate I/R injury in livers with healthy or steatotic parenchyma, but these studies should not include patients with cholestatic livers.

## Abbreviations

ALT: Alanine aminotransferase
ATIII: Antithrombin-III
ATP: Adenosine triphosphate
ATV: Atorvastatin
BDL: Bile duct ligation
CAT: Catalase
DAMP: Damage-associated molecular pattern
DMSO: Dimethyl sulfoxide
eNOS: Endothelial nitric oxide synthase
FOV: Fields of view
GPx: Glutathione peroxidase
H&E: Hematoxylin and eosin
HMGB1: High mobility group box 1
HMG-CoA reductase: 3-hydroxy-3-methyl-glutaryl-coenzyme A reductase
i.v.: Intravenous
ICAM-1: Intracellular adhesion molecule-1
IL-6: Interleukin 6
IR: Ischemia/reperfusion
NADPH: Nicotinamide adenine dinucleotide phosphate
NF-κB: Nuclear factor kappa-light-chain-enhancer of activated B cells
NO: Nitric oxide
NOS: Nitric oxide synthase
RNS: Reactive nitrogen species
ROS: Reactive oxygen species
SEM: Standard error of the mean
SOD: Superoxide dismutase
TLR-4: Toll-like receptor-4
TNF-a: Tumor necrosis factor alpha.
